# Supplementary material for: Lung cancer diagnosis using deep attention‐based multiple instance learning and radiomics
Source: Med Phys. 2022 Mar 3;49(5):3134–43. doi: 10.1002/mp.15539 (PMC9310706; doi:10.1002/mp.15539)
Supplement: Supplementary file 1 — Supporting information [file MP-49-3134-s001.docx]

**Supplementary Materials**

**Supplementary Table 1**. Index of available patients for experiments in LIDC-IDRI

| LIDC-IDRI-0068 | LIDC-IDRI-0072 | LIDC-IDRI-0088 | LIDC-IDRI-0090 |
| --- | --- | --- | --- |
| LIDC-IDRI-0091 | LIDC-IDRI-0118 | LIDC-IDRI-0124 | LIDC-IDRI-0129 |
| LIDC-IDRI-0135 | LIDC-IDRI-0137 | LIDC-IDRI-0138 | LIDC-IDRI-0149 |
| LIDC-IDRI-0159 | LIDC-IDRI-0161 | LIDC-IDRI-0162 | LIDC-IDRI-0163 |
| LIDC-IDRI-0164 | LIDC-IDRI-0165 | LIDC-IDRI-0166 | LIDC-IDRI-0167 |
| LIDC-IDRI-0168 | LIDC-IDRI-0169 | LIDC-IDRI-0171 | LIDC-IDRI-0175 |
| LIDC-IDRI-0179 | LIDC-IDRI-0180 | LIDC-IDRI-0181 | LIDC-IDRI-0182 |
| LIDC-IDRI-0183 | LIDC-IDRI-0184 | LIDC-IDRI-0185 | LIDC-IDRI-0186 |
| LIDC-IDRI-0187 | LIDC-IDRI-0188 | LIDC-IDRI-0190 | LIDC-IDRI-0191 |
| LIDC-IDRI-0192 | LIDC-IDRI-0193 | LIDC-IDRI-0194 | LIDC-IDRI-0200 |
| LIDC-IDRI-0203 | LIDC-IDRI-0207 | LIDC-IDRI-0210 | LIDC-IDRI-0220 |
| LIDC-IDRI-0223 | LIDC-IDRI-0233 | LIDC-IDRI-0234 | LIDC-IDRI-0236 |
| LIDC-IDRI-0237 | LIDC-IDRI-0242 | LIDC-IDRI-0244 | LIDC-IDRI-0246 |
| LIDC-IDRI-0247 | LIDC-IDRI-0249 | LIDC-IDRI-0250 | LIDC-IDRI-0252 |
| LIDC-IDRI-0254 | LIDC-IDRI-0255 | LIDC-IDRI-0256 | LIDC-IDRI-0257 |
| LIDC-IDRI-0258 | LIDC-IDRI-0260 | LIDC-IDRI-0264 | LIDC-IDRI-0265 |
| LIDC-IDRI-0266 | LIDC-IDRI-0267 | LIDC-IDRI-0271 | LIDC-IDRI-0273 |
| LIDC-IDRI-0274 | LIDC-IDRI-0276 | LIDC-IDRI-0277 | LIDC-IDRI-0283 |
| LIDC-IDRI-0285 | LIDC-IDRI-0286 | LIDC-IDRI-0289 | LIDC-IDRI-0290 |
| LIDC-IDRI-0314 | LIDC-IDRI-0325 | LIDC-IDRI-0332 | LIDC-IDRI-0377 |
| LIDC-IDRI-0385 | LIDC-IDRI-0399 | LIDC-IDRI-0405 | LIDC-IDRI-0454 |
| LIDC-IDRI-0470 | LIDC-IDRI-0493 | LIDC-IDRI-0510 | LIDC-IDRI-0543 |
| LIDC-IDRI-0559 | LIDC-IDRI-0562 | LIDC-IDRI-0568 | LIDC-IDRI-0580 |
| LIDC-IDRI-0610 | LIDC-IDRI-0624 | LIDC-IDRI-0766 | LIDC-IDRI-0771 |
| LIDC-IDRI-0772 | LIDC-IDRI-0811 | LIDC-IDRI-0875 | LIDC-IDRI-0893 |
| LIDC-IDRI-0905 | LIDC-IDRI-0921 | LIDC-IDRI-0924 | LIDC-IDRI-0939 |
| LIDC-IDRI-0965 | LIDC-IDRI-0994 | LIDC-IDRI-1002 | LIDC-IDRI-1004 |
| LIDC-IDRI-1011 | LIDC-IDRI-0211 |  |  |

**Supplementary Table 2.** Parameters of params.yaml for radiomics extraction

| Parameters | Value |
| --- | --- |
| binWidth | 25 |
| interpolator | 'sitkBSpline' |
| resampledPixelSpacing | [2, 2, 2] |
| padDistance | 10 |
| resegmentRange | [-3, 3] |
| resegmentMode | sigma |
| voxelArrayShift | 1000 |
| label | 1 |

**Supplementary Table 3**. Radiomics features calculated by using Pyradiomics

| Index | Features | Index | Features |
| --- | --- | --- | --- |
| 1 | original_shape_Elongation | 53 | original_shape_Flatness |
| 2 | original_shape_LeastAxisLength | 54 | original_shape_MajorAxisLength |
| 3 | original_shape_Maximum2DDiameterColumn | 55 | original_shape_Maximum2DDiameterRow |
| 4 | original_shape_Maximum2DDiameterSlice | 56 | original_shape_MeshVolume |
| 5 | original_shape_Maximum3DDiameter | 57 | original_shape_MinorAxisLength |
| 6 | original_shape_Sphericity | 58 | original_shape_SurfaceArea |
| 7 | original_firstorder_10Percentile | 59 | original_glrlm_LongRunLowGrayLevelEmphasis |
| 8 | original_firstorder_90Percentile | 60 | original_glrlm_LowGrayLevelRunEmphasis |
| 9 | original_firstorder_Energy | 61 | original_glrlm_RunEntropy |
| 10 | original_firstorder_Entropy | 62 | original_glrlm_RunLengthNonUniformity |
| 11 | original_firstorder_InterquartileRange | 63 | original_glrlm_RunLengthNonUniformityNormalized |
| 12 | original_firstorder_Kurtosis | 64 | original_glrlm_RunPercentage |
| 13 | original_firstorder_Maximum | 65 | original_glrlm_RunVariance |
| 14 | original_firstorder_Mean | 66 | original_glrlm_ShortRunEmphasis |
| 15 | original_firstorder_MeanAbsoluteDeviation | 67 | original_glrlm_ShortRunHighGrayLevelEmphasis |
| 16 | original_firstorder_Median | 68 | original_glrlm_ShortRunLowGrayLevelEmphasis |
| 17 | original_firstorder_Minimum | 69 | original_glszm_GrayLevelNonUniformity |
| 18 | original_firstorder_Range | 70 | original_glszm_GrayLevelNonUniformityNormalized |
| 19 | original_firstorder_RobustMeanAbsoluteDeviation | 71 | original_glszm_GrayLevelVariance |
| 20 | original_firstorder_RootMeanSquared | 72 | original_glszm_HighGrayLevelZoneEmphasis |
| 21 | original_firstorder_Skewness | 73 | original_glszm_LargeAreaEmphasis |
| 22 | original_firstorder_Uniformity | 74 | original_glszm_LargeAreaHighGrayLevelEmphasis |
| 23 | original_firstorder_Variance | 75 | original_glszm_LargeAreaLowGrayLevelEmphasis |
| 24 | original_glcm_Autocorrelation | 76 | original_glszm_LowGrayLevelZoneEmphasis |
| 25 | original_glcm_JointAverage | 77 | original_glszm_SizeZoneNonUniformity |
| 26 | original_glcm_ClusterProminence | 78 | original_glszm_SizeZoneNonUniformityNormalized |
| 27 | original_glcm_ClusterShade | 79 | original_glszm_SmallAreaEmphasis |
| 28 | original_glcm_ClusterTendency | 80 | original_glszm_SmallAreaHighGrayLevelEmphasis |
| 29 | original_glcm_Contrast | 81 | original_glszm_SmallAreaLowGrayLevelEmphasis |
| 30 | original_glcm_Correlation | 82 | original_glszm_ZoneEntropy |
| 31 | original_glcm_DifferenceAverage | 83 | original_glszm_ZonePercentage |
| 32 | original_glcm_DifferenceEntropy | 84 | original_glszm_ZoneVariance |
| 33 | original_glcm_DifferenceVariance | 85 | original_gldm_DependenceEntropy |
| 34 | original_glcm_JointEnergy | 86 | original_gldm_DependenceNonUniformity |
| 35 | original_glcm_JointEntropy | 87 | original_gldm_DependenceNonUniformityNormalized |
| 36 | original_glcm_Imc1 | 88 | original_gldm_DependenceVariance |
| 37 | original_glcm_Imc2 | 89 | original_gldm_GrayLevelNonUniformity |
| 38 | original_glcm_Idm | 90 | original_gldm_GrayLevelVariance |
| 39 | original_glcm_Idmn | 91 | original_gldm_HighGrayLevelEmphasis |
| 40 | original_glcm_Id | 92 | original_gldm_LargeDependenceEmphasis |
| 41 | original_glcm_Idn | 93 | original_gldm_LargeDependenceHighGrayLevelEmphasis |
| 42 | original_glcm_InverseVariance | 94 | original_gldm_LargeDependenceLowGrayLevelEmphasis |
| 43 | original_glcm_MaximumProbability | 95 | original_gldm_LowGrayLevelEmphasis |
| 44 | original_glcm_SumEntropy | 96 | original_gldm_SmallDependenceEmphasis |
| 45 | original_glcm_SumSquares | 97 | original_gldm_SmallDependenceHighGrayLevelEmphasis |
| 46 | original_glrlm_GrayLevelNonUniformity | 98 | original_gldm_SmallDependenceLowGrayLevelEmphasis |
| 47 | original_glrlm_GrayLevelNonUniformityNormalized | 99 | original_ngtdm_Busyness |
| 48 | original_glrlm_GrayLevelVariance | 100 | original_ngtdm_Coarseness |
| 49 | original_glrlm_HighGrayLevelRunEmphasis | 101 | original_ngtdm_Complexity |
| 50 | original_glrlm_LongRunEmphasis | 102 | original_ngtdm_Contrast |
| 51 | original_glrlm_LongRunHighGrayLevelEmphasis | 103 | original_ngtdm_Strength |
| 52 | original_shape_SurfaceVolumeRatio |  |  |

**Supplementary Table 4.** Summary of experimental network

| Layer | Type | Input Size | Output size |
| --- | --- | --- | --- |
| 1 | Input | -- | 103*12 |
| 2 | FC + LReLU(Drop=0.5) | 103*12 | 256*12 |
| 3 | FC + LReLU(Drop=0.5) | 256*12 | 128*12 |
| 4 | FC + LReLU(Drop=0.5) | 128*12 | 64*12 |
| 5 | Attention Layer | 64*12 | 64*1 |
| 6 | FC+ LReLU | 64*1 | 32*1 |
| 7 | Output+ softmax | 32*1 | 1*1 |

FC means Full connected layer; LReLU means the activation function of this layer is LReLU; Drop means dropout rate of this layer is 0.5; softmax means the activation function of output layer is softmax.


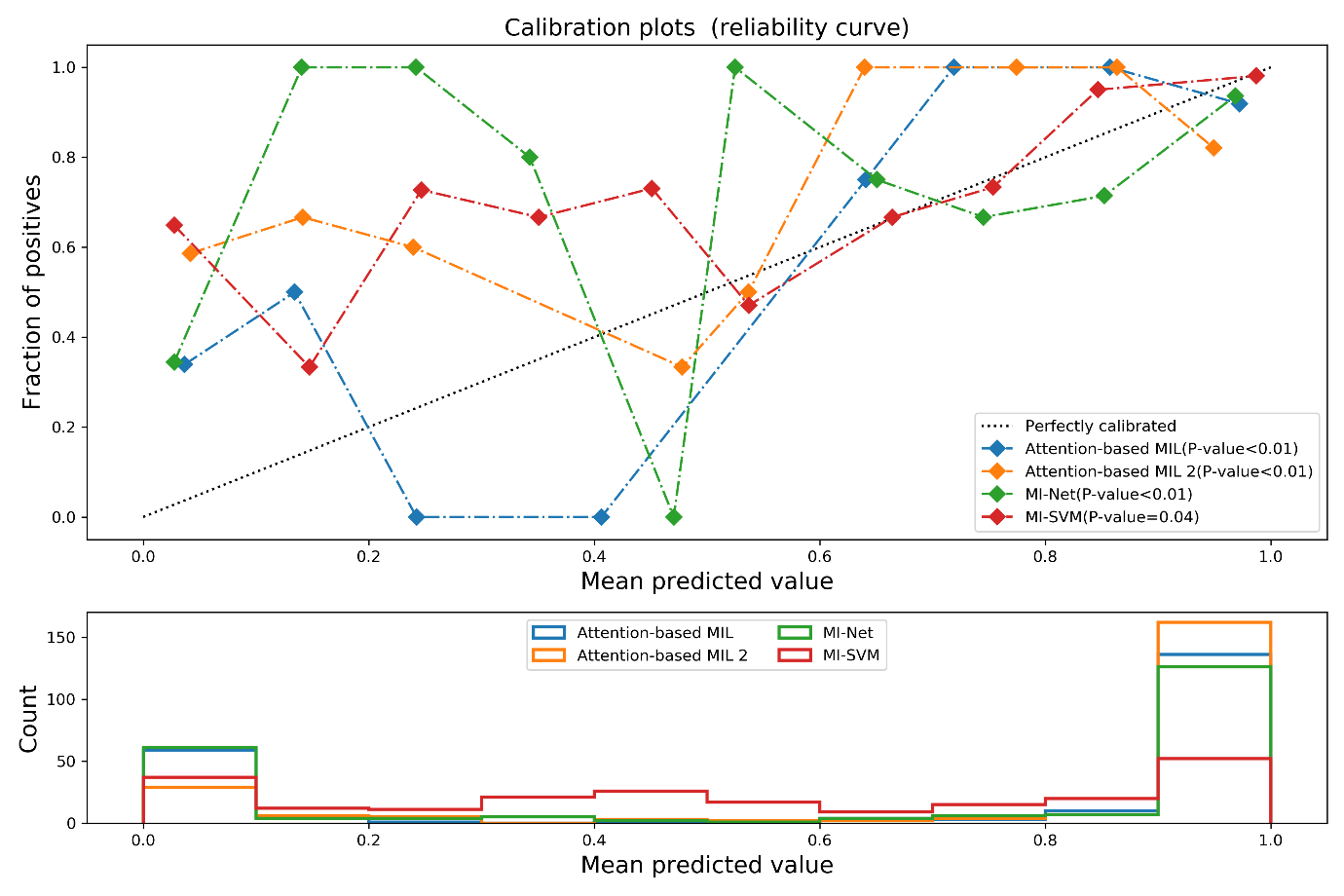


Supplementary Figure 1. Calibration plots and histogram of output probabilities, 10 running results were used in calibration analysis. (Plots of mi-graph and miVLAD absent due to the absent of output probabilities from source code)
